# Hemostatic powder for the management of hemorrhage from esophageal self-expanding metal stents: A case report

**DOI:** 10.1055/a-2302-4686

**Published:** 2024-05-07

**Authors:** Ana Paula Samy Tanaka Kotinda, Antoine Guilloux, Sylvie Breton, Romain Leenhardt, Ulriikka Chaput, Xavier Dray, Marine Camus Duboc

**Affiliations:** 1 Centre for Digestive Endoscopy, Sorbonne University, Saint-Antoine Hospital, APHP, Paris, France; 2 Gastrointestinal Endoscopy Unit – Gastroenterology Department, Hospital das Clínicas da Faculdade de Medicina da Universidade de São Paulo, São Paulo, Brazil; 3 Hepatology Unit, Saint-Antoine Hospital, APHP, Paris, France; 4 Digestive Surgery Unit, Sorbonne University, Pitié Salpétrière Hospital, APHP, Paris, France

Esophageal stenting is a well-established treatment modality for managing various esophageal pathologies. However, despite its benefits, stent-related complications can occur. We present a rare case of hemorrhage following the placement of a fully covered esophageal stent. The prompt identification and management of this complication resulted in a favorable outcome.

We present a case of a 73-year-old man with a complex medical history of esophageal stenosis secondary to esophageal perforation caused by a dental foreign body. Additionally, the patient was under full anticoagulation due to chronic atrial fibrillation. The patient underwent regular follow-up, receiving hydrostatic dilations. Despite partial response to treatment after five dilations, the patient continued to experience dysphagia. A contrast study revealed a stenosis affecting the distal 15 cm of the esophagus. Subsequently, a fully covered esophageal stent measuring 24 × 120 mm was successfully placed without complications. However, on the 8th day post-stent placement, the patient presented with hematemesis, melena, and hemorrhagic shock.

Esophagogastroduodenoscopy revealed entrapment of the stent in the esophageal mucosa and the presence of multiple clots in the esophageal lumen, without active bleeding ( Fig. 1 ). Prompt removal of the stent exposed diffuse and active mucosal bleeding ( Fig. 2 ), which was effectively managed using Nexpowder hemostatic powder (Medtronic, Minneapolis, Minnesota, USA) ( Fig. 3 , Video 1 ). Fluoroscopy confirmed the absence of strictures post-hemostasis. The patient had an uneventful recovery and remained free from further bleeding episodes and dysphagia.

**Fig. 1:**
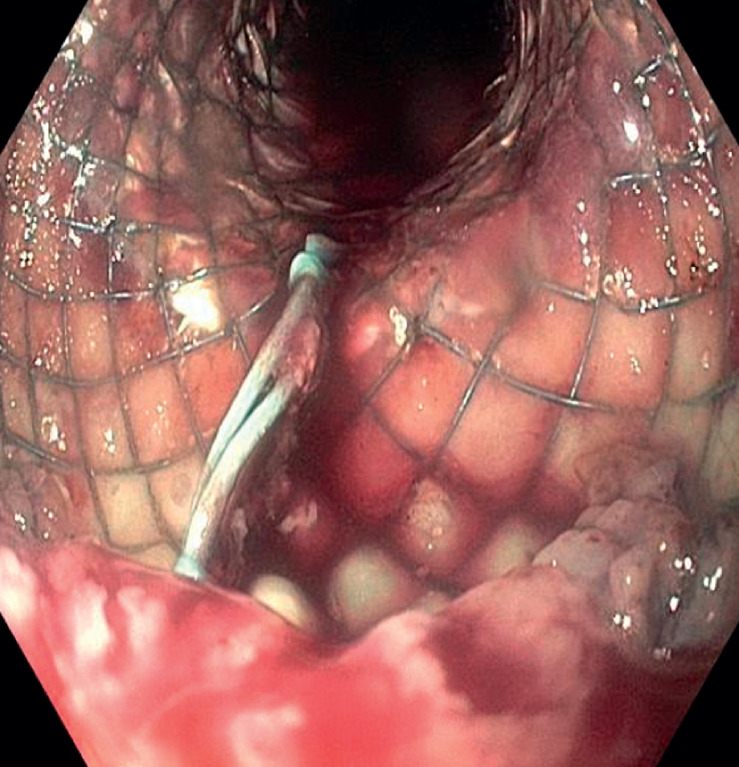
An endoscopic view of the esophagus reveals a self-expanding metal stent with the presence of blood clots within its lumen.

**Fig. 2:**
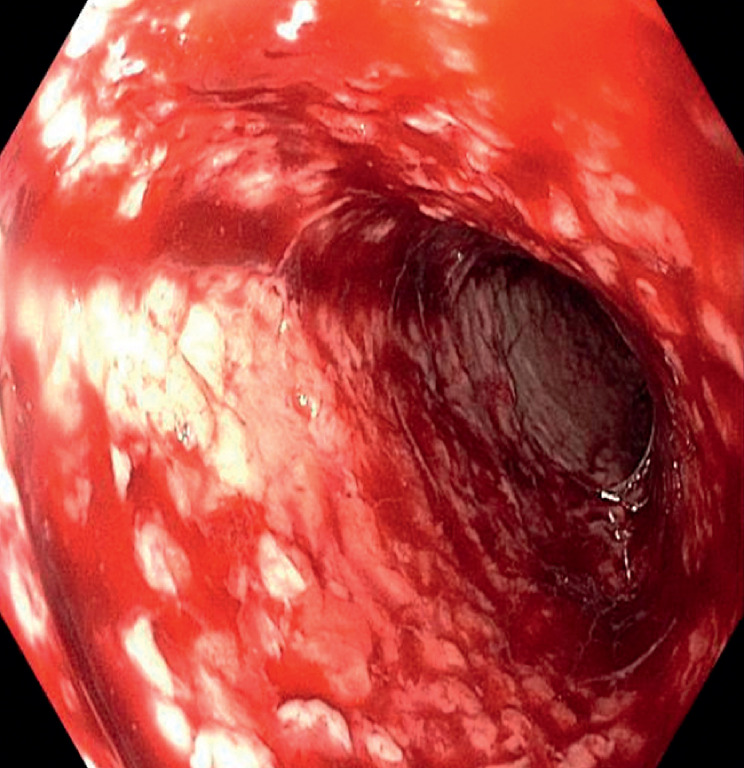
Following the removal of the self-expanding stent, an endoscopic view of the esophagus reveals diffuse bleeding and oozing from the esophageal mucosa.

**Fig. 3:**
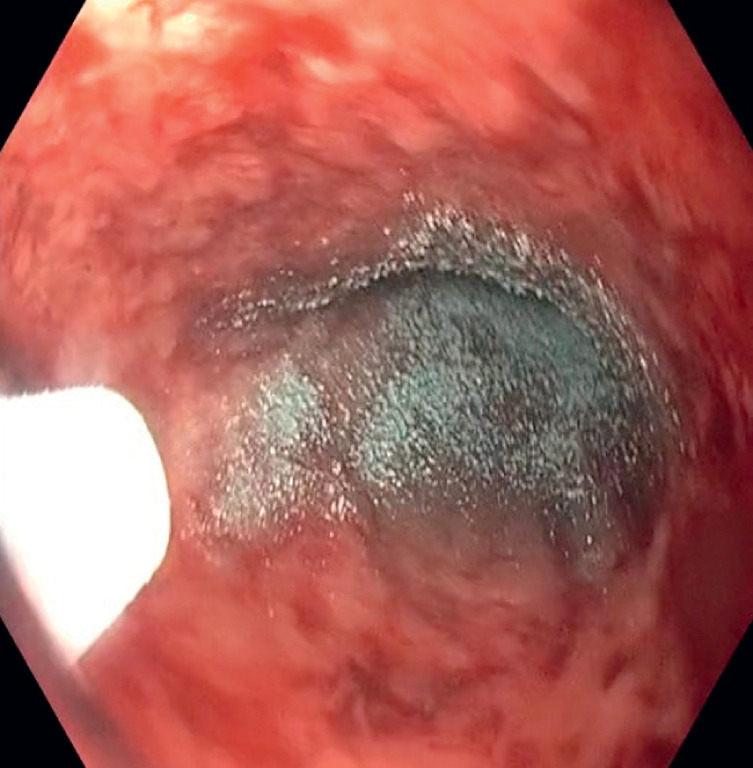
Endoscopic image showcasing the application of Nexpowder hemostatic powder on the site of diffuse esophageal bleeding.

Use of hemostatic powder to manage hemorrhage from esophageal self-expanding metal stent.Video 1

Esophageal stents can be used for the treatment of refractory benign esophageal strictures for a maximum period of 3 months following insertion. The use of self-expanding metal stents as a first-line therapy is not recommended due to the potential for adverse events, the availability of alternative therapies, and the associated cost 1 .

Hemorrhage is a rare but potentially life-threatening complication following esophageal stent placement. Timely recognition and intervention are crucial to ensure optimal patient outcomes. This case highlights the importance of close monitoring post-stenting and the need for prompt endoscopic evaluation in the presence of bleeding symptoms. The Nexpowder endoscopic hemostatic system is a powder used for hemostasis, delivered through a catheter. It is made of a hydrophilic and biocompatible adhesive material that contains succinic anhydride and oxidized dextran. When it comes into contact with water or blood, the system forms an adhesive gel by reversible bonding. This crosslinked gel helps prevent upper gastrointestinal bleeding, fluid loss, and contamination of ulcer sites by adhering to the bleeding site in the upper gastrointestinal tract. Over time, the gel naturally degrades within a period of 1 to 3 days. In our patient's case, the use of hemostatic powder proved to be effective in achieving hemostasis.

Endoscopy_UCTN_Code_CPL_1AH_2AC

## Notes

Endoscopy E-Videos https://eref.thieme.de/e-videos
*E-Videos* is an open access online section of the journal *Endoscopy* , reporting on interesting cases and new techniques in gastroenterological endoscopy. All papers include a high-quality video and are published with a Creative Commons CC-BY license. Endoscopy E-Videos qualify for HINARI discounts and waivers and eligibility is automatically checked during the submission process. We grant 100% waivers to articles whose corresponding authors are based in Group A countries and 50% waivers to those who are based in Group B countries as classified by Research4Life (see: https://www.research4life.org/access/eligibility/ ). This section has its own submission website at https://mc.manuscriptcentral.com/e-videos .

## References

[1] Spaander MCW van der Bogt RD Baron TH Esophageal stenting for benign and malignant disease: European Society of Gastrointestinal Endoscopy (ESGE) Guideline – Update 2021 Endoscopy 2021 53 751 762 10.1055/a-1475-0063 33930932

